# Association of Clinical Staging With MRI Findings Pre and Post Intra-articular Steroid Injections in Patients Suffering From Adhesive Capsulitis: A Prospective Single-Arm Interventional Study

**DOI:** 10.7759/cureus.115028

**Published:** 2026-08-23

**Authors:** Nilesh K Meena, Maheshwar Lakkireddy, Abhishek J Arora, Ranjith K Yalamanchili, Syed Ifthekar, Deepak Kumar Maley, Deepankar Satapathy, Srikanth Eppakayala, Vijay Uppala, Nikhil Lath

**Affiliations:** 1 Orthopedics, All India Institute of Medical Sciences, Bibinagar, Hyderabad, IND; 2 Radiodiagnosis, All India Institute of Medical Sciences, Bibinagar, Hyderabad, IND; 3 Orthopedic Surgery, All India Institute of Medical Sciences, Bibinagar, Hyderabad, IND; 4 Orthopedics and Trauma, All India Institute of Medical Sciences, Bibinagar, Hyderabad, IND

**Keywords:** adhesive capsulitis, clinical staging, frozen shoulder, frozen shoulder steroid instillation, idiopathic adhesive capsulitis, intra-articular steroid injection, mri, primary frozen shoulder, steroid use

## Abstract

Background: Adhesive capsulitis is characterized by progressive capsular inflammation and fibrosis resulting in pain and restriction of shoulder movement. Although clinical staging remains the cornerstone of disease assessment, magnetic resonance imaging (MRI) provides objective information regarding structural changes that may complement clinical evaluation. This study aimed to evaluate the association between clinical staging and MRI findings and to assess clinical and radiological changes following intra-articular corticosteroid injection.

Aim: The primary objective of this study was to evaluate the association between clinical staging and MRI findings in patients with adhesive capsulitis. The secondary objective was to assess changes in clinical, functional, and MRI parameters following intra-articular corticosteroid injection.

Methods: This prospective single-arm interventional study included 19 patients aged 18-70 years diagnosed with adhesive capsulitis at All India Institute of Medical Sciences (AIIMS), Bibinagar. Clinical staging and range of motion were assessed. The following seven MRI parameters were evaluated: (1) thickness of the inferior glenohumeral ligament (IGHL) anterior band (humeral aspect), (2) thickness of the IGHL anterior band (glenoid aspect), (3) thickness of the axillary recess (humeral aspect), (4) thickness of the axillary recess (glenoid aspect), (5) coracohumeral ligament thickness, (6) anterior capsule thickness, and (7) obliteration of the subcoracoid fat triangle were assessed at baseline and after 12-14 weeks following intra-articular triamcinolone injection. Statistical analysis was performed using SPSS version 26 (IBM Corp., Armonk, New York, USA), with p < 0.05 considered significant.

Results: Nineteen patients with adhesive capsulitis were evaluated. Baseline MRI demonstrated progressive thickening of the anterior band of the inferior glenohumeral ligament (IGHL), axillary recess, and anterior capsule, with increasing clinical stage. Significant stage-wise differences were observed in MRI parameters, particularly in the IGHL anterior band and anterior capsule thickness (p < 0.05). Following intra-articular corticosteroid injection, significant improvements were observed in pain (Visual Analogue Scale (VAS)), functional outcome (Disabilities of the Arm, Shoulder and Hand (DASH)), shoulder range of motion, and MRI measurements, indicating both clinical and radiological improvement.

Conclusion: MRI findings correlate significantly with clinical staging and demonstrate measurable structural improvement following intra-articular steroid injection. MRI serves as a valuable tool for staging, monitoring treatment response, and guiding individualized management in adhesive capsulitis.

## Introduction

Adhesive capsulitis (AC) is a frequent, disabling disorder characterized by progressive shoulder pain with global restriction of both active and passive range of motion, ultimately impairing daily function and quality of life [1,2].

Epidemiologically, it affects around 2-5% of the adult population [3] and is over-represented among individuals with metabolic and endocrine comorbidities, with a slight female predominance [4].

The clinical course is typically prolonged, evolving over months, and the overlap of symptoms with other painful shoulder conditions makes it challenging to determine the appropriate clinical stage for diagnosis and management [5,6].

Pathologically, AC is understood as an inflammatory spectrum that begins with synovitis and capsular irritation and progresses toward capsular thickening, contraction, and fibrosis; thus, explaining why pain is often prominent early, while stiffness dominates later [7,8]. Clinically, Neviaser's staged concept and subsequent arthroscopic modifications emphasized that AC is not a single static entity [9,10]; rather, it evolves through distinct stages with differing dominant pathology, an insight that underpins modern treatment algorithms [2,11].

In many clinical settings, AC is diagnosed on history and examination, but real-world practice often involves primary care and non-specialist evaluation, where imaging is frequently used to exclude mimics or concomitant pathology [12-14]. Imaging becomes especially relevant because the clinical picture of AC can resemble calcific tendinitis, impingement syndromes, early glenohumeral osteoarthritis, or rotator cuff disease, and secondary AC may be obscured by the primary shoulder disorder [2,15]. While radiographs are generally normal, magnetic resonance imaging (MRI) offers a non-invasive window into the synovium capsule and structures that are central to AC pathology and that are not directly measurable by physical examination [16-19].

However, despite this expanding use, the literature has highlighted an ongoing lack of consensus on which MRI findings are most clinically meaningful and how consistently they map to clinical stage and impairment, limiting the full integration of MRI into staging-based therapeutic planning [15].

Although adhesive capsulitis is primarily diagnosed and staged clinically, MRI provides objective visualization of capsular inflammation, ligamentous thickening, and structural changes associated with disease progression. Evaluating the association between clinical staging and MRI findings is clinically important because it helps determine whether radiological abnormalities accurately reflect disease severity and whether these imaging findings change in parallel with clinical improvement following intra-articular corticosteroid injection. Establishing this relationship may improve disease assessment, facilitate treatment monitoring, and enhance clinical decision-making.

The present study aims to explore the correlation between the clinical staging system adapted from Hannafin and Chiaia and MRI findings in patients with AC and MRI findings at 12-14 weeks after intra-articular steroid injection by assessing MRI at diagnosis and again 12 to 14 weeks after the injection [20,21]. By systematically correlating stage-based clinical impairment with reproducible MRI parameters both before and after injection, this study seeks to improve stage-specific understanding of adhesive capsulitis, distinguish MRI indicators of active inflammation from features of established fibrosis, and promote more individualized, evidence-aligned management of frozen shoulder.

## Materials and methods

Study design and methodology

This prospective study was conducted in the Department of Orthopedics at the All India Institute of Medical Sciences (AIIMS), Bibinagar, a tertiary care teaching hospital, between October 2023 and October 2025. The study was undertaken to evaluate the correlation between the clinical staging system adapted from Hannafin and Chiaia and magnetic resonance imaging (MRI) findings in patients with adhesive capsulitis who were treated with intra-articular steroid injections at the time of diagnosis and reassessed after 12-14 weeks.

Clinical staging was performed using an adapted Hannafin and Chiaia staging system, which classifies adhesive capsulitis into four stages based on the duration of symptoms, severity of pain, restriction of shoulder range of motion, and clinical progression. The adaptation was made to facilitate standardized clinical staging in routine practice while preserving the fundamental principles of the original classification. Patients in the present study were classified according to these predefined clinical criteria before treatment.

Clinical staging, pain assessment (Visual Analogue Scale (VAS)), functional assessment (Disabilities of the Arm, Shoulder and Hand (DASH)), and shoulder range-of-motion measurements (forward flexion, extension, abduction, external rotation, and internal rotation) were measured using a universal goniometer by the principal investigator at baseline and follow-up.

MRI was performed using a 3-Tesla scanner (Siemens Healthineers, Erlangen, Germany). The IGHL, axillary recess, coracohumeral ligament, and anterior capsule thickness were measured at their maximal thickness using standard anatomical landmarks. Obliteration of the subcoracoid fat triangle was assessed qualitatively. All MRI measurements were performed by an experienced radiologist. As this was a prospective single-arm interventional study, assessor blinding was not performed.

As all enrolled participants received the same intervention without randomization or a comparison group, the study was designed as a single-arm interventional clinical study.

Study site

The study was carried out in the Outpatient Department of Orthopedics, AIIMS Bibinagar.

Study population

The study population comprised male and female patients aged 18-70 years presenting to the Orthopedics Outpatient Department with a clinical diagnosis of adhesive capsulitis (clinical stages 1-3). Patients who provided written informed consent for intra-articular steroid injection and clinico-radiological evaluation were included and evaluated during the study period.

Study duration phases

The study was conducted over 24 months from October 2023 to October 2025. This period included patient recruitment, administration of intra-articular steroid injections, scheduled follow-up visits, MRI evaluation at 12-14 weeks, data analysis, and manuscript preparation.

Method of recruitment

Patients attending the Orthopedics Outpatient Department at AIIMS Bibinagar with symptoms suggestive of adhesive capsulitis were screened clinically. Eligible patients were staged using the clinical staging system adapted from Hannafin and Chiaia [22].

Those fulfilling the inclusion criteria were advised to undergo an MRI of the shoulder joint. After confirmation, an intra-articular steroid injection was administered, and patients were followed up at four-weekly intervals. A repeat clinical assessment and MRI examination were performed after 12-14 weeks.

Sampling method

A convenience sampling technique was employed. Eligible patients attending the Orthopedics Outpatient Department who fulfilled the inclusion and exclusion criteria and provided written informed consent were prospectively recruited during the study period until the target sample size was achieved. Consecutive enrollment of all eligible patients was not performed due to the predefined study duration and practical recruitment constraints.

Selection criteria

Patients were selected based on the following criteria: Patients with adhesive capsulitis corresponding to clinical stages 1-3 according to the Hannafin and Chiaia clinical staging system [22] were included in the study. Provision of written informed consent to participate in the study.

Inclusion criteria: Patients aged 18-70 years diagnosed with adhesive capsulitis corresponding to clinical stages 1-3 according to the Hannafin and Chiaia classification and who provided written informed consent were included in the study. The age range of 18-70 years was selected to represent the adult population with adhesive capsulitis. To maintain a homogeneous study population, only patients with primary adhesive capsulitis were included, while secondary causes of shoulder stiffness were excluded.

Exclusion criteria: Patients with pre-existing shoulder pathology, post-traumatic shoulder stiffness, previous shoulder injury, or a history of cerebrovascular accident affecting the involved limb were excluded from the study.

Study procedure

Eligible patients were explained the purpose and methodology of the study, and written informed consent was obtained. A detailed clinical assessment was performed, and patients were staged using the clinical staging system adapted from Hannafin and Chiaia. Blood samples were collected for random blood sugar and lipid profile estimation.

An MRI of the shoulder without contrast was performed for all patients. The following seven MRI parameters were evaluated: (1) thickness of the IGHL anterior band (humeral aspect), (2) thickness of the IGHL anterior band (glenoid aspect), (3) thickness of the axillary recess (humeral aspect), (4) thickness of the axillary recess (glenoid aspect), (5) coracohumeral ligament thickness, (6) anterior capsule thickness, and (7) obliteration of the subcoracoid fat triangle.

Following MRI evaluation, patients received an intra-articular steroid injection consisting of triamcinolone 10 mg combined with a local anesthetic, administered via the posterior approach without ultrasound guidance, using anatomical bony landmarks. Patients were advised to continue non-opioid analgesics for symptomatic relief and to perform shoulder range-of-motion exercises for 12 weeks. Following intra-articular corticosteroid injection, patients were advised to perform a standardized home-based exercise program, including pendulum, stretching, wall-climbing, and active-assisted range-of-motion exercises, with progression according to clinical improvement at follow-up.

Pain intensity was assessed using the Visual Analogue Scale (VAS). Functional outcome was evaluated using the Disabilities of the Arm, Shoulder and Hand (DASH) questionnaire, a validated patient-reported outcome measure developed by Hudak et al. to assess upper-extremity disability and symptoms [23]. Follow-up assessments were conducted at four-weekly intervals. At 12-14 weeks, repeat clinical staging and MRI evaluation were performed. All collected data were used to establish a clinico-radiological correlation.

Sample size

The sample size was calculated using the formula:

$n =\left( Z^{2} \times p \times\left( 1-p \right) \right)/e^{2}$

n = ((1.96)² × 0.05 × (1 − 0.05))/(0.10)²

n = (3.8416 × 0.05 × 0.95)/0.01

n = 18.25 ≈ 19

Therefore, the minimum required sample size was 19 participants. The sample size was estimated using a prevalence-based formula because reliable published data were not available to estimate an effect size for a formal power analysis at the time of study design. No a priori power analysis was performed, and the study should be considered exploratory.

Statistical analysis

Statistical analysis was performed using SPSS version 23.0 (IBM Corp., Armonk, NY, USA). Continuous variables were expressed as mean ± standard deviation, and categorical variables as frequencies and percentages. Baseline MRI parameters were compared among the three clinical stages (Stage 1, Stage 2, and Stage 3) using one-way analysis of variance (ANOVA), followed by post hoc pairwise comparisons where appropriate. Pre- and post-intervention continuous variables were compared using the paired t-test. Categorical variables were analyzed using the Chi-square test. A p-value < 0.05 was considered statistically significant.

Ethical considerations

The study protocol was approved by the Institutional Ethics Committee before the initiation of patient recruitment at All India Institute of Medical Sciences (AIIMS), Bibinagar, under approval number AIIMS/BBN/IEC/NOV/2023/349. The study was conducted in accordance with the ethical principles of the Declaration of Helsinki. Written informed consent was obtained from all participants prior to enrollment. Confidentiality and anonymity of patient data were maintained throughout the study. Participation was voluntary, and patients were informed of their right to withdraw from the study at any stage without affecting their treatment.

This was an investigator-initiated, single-arm interventional study approved by the Institutional Ethics Committee. Clinical trial registration was not obtained.

## Results

A total of 19 patients with adhesive capsulitis fulfilling the inclusion criteria were enrolled in this prospective observational study. The baseline demographic characteristics, laboratory parameters, clinical scores, range of motion, and MRI findings were analyzed before treatment and reassessed 12-14 weeks following intra-articular steroid injection.

The study population predominantly consisted of patients aged 51-60 years (47.37%), followed by those aged 41-50 years (31.58%). Males constituted 63.16% of the study population, while females accounted for 36.84%. Left shoulder involvement was slightly more common than right shoulder involvement. The demographic characteristics of the study population are summarized in Tables 1-3. 

**Table 1:** Age distribution.

Age	Frequency	Percentage
31-40 years	1	5.26
41-50 years	6	31.58
51-60 years	9	47.37
61-70 years	3	15.79
Total	19	100.00

**Table 2:** Gender distribution.

Gender	Frequency	Percentage
Male	12	63.16
Female	7	36.84

**Table 3:** Side of limb involvement.

Limb involved	Frequency	Percentage
Right	9	47.37
Left	10	52.63

Baseline laboratory investigations demonstrated mildly elevated mean total cholesterol and triglyceride levels, while the average random blood sugar was within acceptable limits (Table 4). 

**Table 4:** Baseline laboratory parameters. RBS: random blood sugar; TC: total cholesterol; TG: triglycerides; HDL: high-density lipoprotein; LDL: low-density lipoprotein.

Lab parameters	Mean	SD
RBS	115.68	17.32
TC	215.95	40.29
LDL	123.89	36.28
HDL	54.37	12.29
TG	192.89	48.84

Pain and functional disability improved progressively throughout follow-up. Mean VAS scores decreased from 7.68 before injection to 3.11 at 12 weeks, whereas mean DASH scores improved from 34.93 to 14.61 over the same period (Tables 5, 6).

**Table 5:** DASH scores over follow-up. DASH: Disabilities of the Arm, Shoulder and Hand.

DASH score	Pre-injection	4 Weeks	8 Weeks	12 Weeks
Mean	34.93	27.97	18.71	14.61
SD	13.52	10.83	10.49	10.16

**Table 6:** VAS scores over follow-up. VAS: Visual Analogue Scale.

VAS score	Pre-injection	4 Weeks	8 Weeks	12 Weeks
Mean	7.68	6	4.42	3.11
SD	1.057	1	1.61	1.696

At baseline, the majority of patients were classified as Stage 2 adhesive capsulitis (57.89%), followed by Stage 3 (31.58%), while only 10.53% of patients were in Stage 1, indicating that most patients presented during the painful stiffening phase of the disease (Table 7). 

**Table 7:** Clinical stage distribution at baseline.

Hannafin Chiaia clinical stage (pre-injection)	Frequency	Percentage
Stage 1	2	10.53
Stage 2	11	57.89
Stage 3	6	31.58

Baseline shoulder range of motion differed significantly across the clinical stages. Internal rotation, external rotation, abduction, adduction, flexion, and extension all demonstrated statistically significant differences among the stages (p < 0.05), with Stage 2 patients exhibiting the greatest restriction of movement (Table 8).

**Table 8:** Baseline range of motion across clinical stages. Statistical analysis: one-way ANOVA.

Movement (pre-injection)	Stage	Mean ± SD	F-value	p-value
Internal rotation	Stage 1 (n=2)	35.00 ± 0.00	44.401	<0.001
Internal rotation	Stage 2 (n=11)	28.18 ± 4.62	44.401	<0.001
Internal rotation	Stage 3 (n=6)	51.67 ± 9.83	44.401	<0.001
Total	(n=19)	38.16 ± 8.85	44.401	<0.001
External rotation	Stage 1 (n=2)	25.00 ± 7.07	5.015	0.02
External rotation	Stage 2 (n=11)	16.36 ± 4.52	5.015	0.02
External rotation	Stage 3 (n=6)	38.33 ± 4.08	5.015	0.02
Total	(n=19)	24.21 ± 11.09	5.015	0.02
Abduction	Stage 1 (n=2)	60.00 ± 7.07	17.836	<0.001
Abduction	Stage 2 (n=11)	36.82 ± 6.81	17.836	<0.001
Abduction	Stage 3 (n=6)	78.33 ± 9.83	17.836	<0.001
Total	(n=19)	36.32 ± 12.57	17.836	<0.001
Adduction	Stage 1 (n=2)	12.50 ± 3.54	24.622	<0.001
Adduction	Stage 2 (n=11)	11.82 ± 2.52	24.622	<0.001
Adduction	Stage 3 (n=6)	19.17 ± 2.04	24.622	<0.001
Total	(n=19)	14.21 ± 4.17	24.622	<0.001
Flexion	Stage 1 (n=2)	42.50 ± 3.54	40.113	<0.001
Flexion	Stage 2 (n=11)	33.64 ± 7.78	40.113	<0.001
Flexion	Stage 3 (n=6)	45.00 ± 7.07	40.113	<0.001
Total	(n=19)	52.37 ± 20.84	40.113	<0.001
Extension	Stage 1 (n=2)	17.50 ± 3.54	54.737	<0.001
Extension	Stage 2 (n=11)	12.73 ± 2.61	54.737	<0.001
Extension	Stage 3 (n=6)	24.17 ± 2.04	54.737	<0.001
Total	(n=19)	16.84 ± 5.82	54.737	<0.001

Baseline MRI measurements demonstrated significant differences among the clinical stages for the anterior band of the inferior glenohumeral ligament (IGHL), axillary recess thickness, and anterior capsule thickness (p < 0.05). However, coracohumeral ligament thickness did not differ significantly between stages (p=0.122) (Table 9).

**Table 9:** Baseline MRI parameters across clinical stages. Statistical analysis: one-way ANOVA. IGHL: inferior glenohumeral ligament.

Parameter (pre-injection)	Stage	Mean ± SD	F-value	p-value
IGHL anterior band (humeral aspect)	Stage 1 (n=2)	4.50 ± 0.14	21.73	<0.001
IGHL anterior band (humeral aspect)	Stage 2 (n=11)	8.03 ± 0.68	21.73	<0.001
IGHL anterior band (humeral aspect)	Stage 3 (n=6)	6.70 ± 0.89	21.73	<0.001
Total	(n=19)	7.24 ± 1.34	21.73	<0.001
IGHL anterior band (glenoid aspect)	Stage 1 (n=2)	4.35 ± 0.21	14.847	<0.001
IGHL anterior band (glenoid aspect)	Stage 2 (n=11)	7.72 ± 0.79	14.847	<0.001
IGHL anterior band (glenoid aspect)	Stage 3 (n=6)	6.60 ± 0.99	14.847	<0.001
Total	(n=19)	7.01 ± 1.33	14.847	<0.001
Axillary recess (humeral aspect)	Stage 1 (n=2)	4.10 ± 0.71	9.804	0.002
Axillary recess (humeral aspect)	Stage 2 (n=11)	5.72 ± 0.90	9.804	0.002
Axillary recess (humeral aspect)	Stage 3 (n=6)	4.10 ± 0.50	9.804	0.002
Total	(n=19)	5.04 ± 1.11	9.804	0.002
Axillary recess (glenoid aspect)	Stage 1 (n=2)	3.75 ± 0.64	9.286	0.002
Axillary recess (glenoid aspect)	Stage 2 (n=11)	5.50 ± 1.00	9.286	0.002
Axillary recess (glenoid aspect)	Stage 3 (n=6)	3.80 ± 0.51	9.286	0.002
Total	(n=19)	4.78 ± 1.19	9.286	0.002
Coracohumeral ligament	Stage 1 (n=2)	1.80 ± 0.00	2.41	0.122
Coracohumeral ligament	Stage 2 (n=11)	1.99 ± 0.50	2.41	0.122
Coracohumeral ligament	Stage 3 (n=6)	2.55 ± 0.70	2.41	0.122
Total	(n=19)	2.15 ± 0.60	2.41	0.122
Anterior capsule thickness	Stage 1 (n=2)	3.75 ± 0.07	26.017	<0.001
Anterior capsule thickness	Stage 2 (n=11)	5.16 ± 0.30	26.017	<0.001
Anterior capsule thickness	Stage 3 (n=6)	4.37 ± 0.33	26.017	<0.001
Total	(n=19)	4.76 ± 0.59	26.017	<0.001

Subcoracoid fat triangle obliteration was observed across all clinical stages at baseline. Although the proportion of patients with fat triangle obliteration was highest in Stage 3, the overall distribution did not differ significantly among the clinical stages (χ²=0.707, p=0.702) (Table 10).

**Table 10:** Subcoracoid fat triangle obliteration at baseline. Statistical analysis: chi-square test.

Obliteration of the subcoracoid fat triangle (pre-injection)	Stage 1	Stage 1	Stage 2	Stage 2	Stage 3	Stage 3
Number and percentage	N	%	N	%	N	%
Yes	1	50.00%	5	45.45%	4	66.67%
No	1	50.00%	6	54.55%	2	33.33%
Chi-square value	0.707	0.707	0.707	0.707	0.707	0.707
p-value	0.702	0.702	0.702	0.702	0.702	0.702

Pairwise comparison of baseline MRI parameters demonstrated significant differences in the thickness of the anterior band of the inferior glenohumeral ligament and anterior capsule between most stage comparisons (p < 0.05). Significant differences were also observed for axillary recess thickness between selected stages, whereas coracohumeral ligament thickness showed no significant pairwise differences (Table 11).

**Table 11:** Baseline pairwise comparison of MRI parameters. Statistical analysis: Independent samples t-test with post-hoc pairwise comparisons. IGHL: inferior glenohumeral ligament.

Variable (pre-injection)	Stage 1 vs 2		Stage 2 vs 3		Stage 1 vs 3	
	t-value	p-value	t-value	p-value	t-value	p-value
IGHL anterior band-humeral	-7.033	<0.001	3.442	0.004	-3.292	0.017
IGHL anterior band-glenoid	-5.798	<0.001	2.562	0.022	-3.048	0.023
Axillary recess-humeral	-2.373	0.037	4.028	0.001	<0.001	1
Axillary recess-glenoid	-2.343	0.039	3.859	0.002	-0.114	0.913
Coracohumeral ligament	-0.522	0.612	-1.923	0.074	-1.442	0.199
Anterior capsule thickness	-6.323	<0.001	5.035	<0.001	-2.521	0.045

At the 12-week follow-up, the clinical stage distribution demonstrated a marked shift toward recovery. Most patients had progressed to Stage 4 (57.89%), while 31.58% remained in Stage 3 and only 10.53% remained in Stage 2, indicating substantial clinical improvement following intra-articular steroid injection (Table 12).

**Table 12:** Clinical stage distribution at 12 weeks. Statistical analysis: Independent samples t-test with post-hoc pairwise comparisons.

Hannafin Chiaia clinical stage (12 weeks)	Frequency	Percentage
Stage 2	2	10.53
Stage 3	6	31.58
Stage 4	11	57.89

At the 12-week follow-up, shoulder range of motion improved significantly across all clinical stages. Patients in Stage IV demonstrated the greatest improvement in internal rotation, external rotation, abduction, adduction, flexion, and extension, whereas patients remaining in Stage 2 continued to exhibit comparatively restricted movements. Significant differences in all range-of-motion parameters were observed among the clinical stages (Table 13).

**Table 13:** Range of motion at 12 weeks across stages. Statistical analysis: One-way ANOVA.

Movement (12 weeks)	Stage	Mean ± SD	F-value	p-value
Internal rotation	Stage 2 (n=2)	35.00 ± 7.07	42.118	<0.001
Internal rotation	Stage 3 (n=6)	56.67 ± 2.58	42.118	<0.001
Internal rotation	Stage 4 (n=11)	66.82 ± 5.14	42.118	<0.001
Total	(n=19)	60.26 ± 10.99	42.118	<0.001
External rotation	Stage 2 (n=2)	22.50 ± 3.54	62.659	<0.001
External rotation	Stage 3 (n=6)	45.00 ± 4.47	62.659	<0.001
External rotation	Stage 4 (n=11)	69.55 ± 7.23	62.659	<0.001
Total	(n=19)	56.84 ± 17.66	62.659	<0.001
Abduction	Stage 2 (n=2)	30.00 ± 0.00	36.163	<0.001
Abduction	Stage 3 (n=6)	61.67 ± 12.52	36.163	<0.001
Abduction	Stage 4 (n=11)	85.00 ± 7.42	36.163	<0.001
Total	(n=19)	71.84 ± 20.22	36.163	<0.001
Adduction	Stage 2 (n=2)	10.00 ± 0.00	31.392	<0.001
Adduction	Stage 3 (n=6)	23.33 ± 4.08	31.392	<0.001
Adduction	Stage 4 (n=11)	28.18 ± 2.52	31.392	<0.001
Total	(n=19)	24.74 ± 6.34	31.392	<0.001
Flexion	Stage 2 (n=2)	45.00 ± 7.07	55.275	<0.001
Flexion	Stage 3 (n=6)	89.17 ± 10.69	55.275	<0.001
Flexion	Stage 4 (n=11)	111.82 ± 7.51	55.275	<0.001
Total	(n=19)	97.63 ± 22.81	55.275	<0.001
Extension	Stage 2 (n=2)	12.50 ± 3.54	30.93	<0.001
Extension	Stage 3 (n=6)	31.67 ± 4.08	30.93	<0.001
Extension	Stage 4 (n=11)	36.36 ± 3.93	30.93	<0.001
Total	(n=19)	32.37 ± 8.23	30.93	<0.001

MRI evaluation performed at 12 weeks demonstrated a reduction in capsular thickness compared with baseline. Significant differences among clinical stages were observed for the IGHL anterior band at the glenoid aspect and anterior capsule thickness, whereas differences in the remaining MRI parameters were not statistically significant (Table 14).

**Table 14:** MRI parameters at 12 weeks across stages. IGHL: inferior glenohumeral ligament.

Parameter (12 weeks)	Stage	Mean ± SD	F-value	p-value
IGHL anterior band (humeral aspect)	Stage 2 (n=2)	7.20 ± 0.00	2.754	0.094
IGHL anterior band (humeral aspect)	Stage 3 (n=6)	6.33 ± 0.74	2.754	0.094
IGHL anterior band (humeral aspect)	Stage 4 (n=11)	5.94 ± 0.75	2.754	0.094
Total	(n=19)	6.20 ± 0.79	2.754	0.094
IGHL anterior band (glenoid aspect)	Stage 2 (n=2)	6.80 ± 0.42	4.346	0.031
IGHL anterior band (glenoid aspect)	Stage 3 (n=6)	6.10 ± 0.74	4.346	0.031
IGHL anterior band (glenoid aspect)	Stage 4 (n=11)	5.52 ± 0.58	4.346	0.031
Total	(n=19)	5.84 ± 0.73	4.346	0.031
Axillary recess (humeral aspect)	Stage 2 (n=2)	5.20 ± 1.98	0.819	0.458
Axillary recess (humeral aspect)	Stage 3 (n=6)	4.62 ± 0.86	0.819	0.458
Axillary recess (humeral aspect)	Stage 4 (n=11)	4.34 ± 0.75	0.819	0.458
Total	(n=19)	4.52 ± 0.90	0.819	0.458
Axillary recess (glenoid aspect)	Stage 2 (n=2)	5.30 ± 1.56	1.704	0.213
Axillary recess (glenoid aspect)	Stage 3 (n=6)	4.37 ± 0.88	1.704	0.213
Axillary recess (glenoid aspect)	Stage 4 (n=11)	4.13 ± 0.69	1.704	0.213
Total	(n=19)	4.33 ± 0.86	1.704	0.213
Coracohumeral ligament	Stage 2 (n=2)	1.95 ± 0.07	0.045	0.957
Coracohumeral ligament	Stage 3 (n=6)	2.08 ± 0.57	0.045	0.957
Coracohumeral ligament	Stage 4 (n=11)	2.09 ± 0.67	0.045	0.957
Total	(n=19)	2.07 ± 0.59	0.045	0.957
Anterior capsule thickness	Stage 2 (n=2)	4.55 ± 0.64	4.128	0.036
Anterior capsule thickness	Stage 3 (n=6)	4.70 ± 0.45	4.128	0.036
Anterior capsule thickness	Stage 4 (n=11)	4.11 ± 0.37	4.128	0.036
Total	(n=19)	4.34 ± 0.49	4.128	0.036

The frequency of subcoracoid fat triangle obliteration differed significantly among clinical stages at 12 weeks. Obliteration was absent in Stage 2, present in all Stage 3 patients, and observed in the majority of Stage 4 patients (Table 15).

**Table 15:** Subcoracoid fat triangle obliteration at 12 weeks. Statistical analysis: Chi-square test.

Obliteration of the subcoracoid fat triangle (12 weeks)	Stage 2	Stage 2	Stage 3	Stage 3	Stage 4	Stage 4
Number and percentage	N	%	N	%	N	%
Yes	0	0.00%	6	100.00%	9	81.82%
No	2	100.00%	0	0.00%	2	18.18%
Chi-square value	9.155	9.155	9.155	9.155	9.155	9.155
p-value	0.01	0.01	0.01	0.01	0.01	0.01

Pairwise comparison of MRI parameters at 12 weeks demonstrated significant differences in selected imaging parameters between clinical stages. Significant differences were observed for the humeral aspect of the IGHL anterior band between Stages 2 and 3, the glenoid aspect of the IGHL anterior band between Stages 2 and 4, and anterior capsule thickness between Stages 3 and 4 (Table 16).

**Table 16:** Pairwise MRI comparison at 12 weeks. Statistical analysis: Independent samples t-test. IGHL: inferior glenohumeral ligament.

Variable (12 weeks)	Stage 2 vs 3	Stage 2 vs 3	Stage 2 vs 4	Stage 2 vs 4	Stage 3 vs 4	Stage 3 vs 4
t-value and p-value	t-value	p-value	t-value	p-value	t-value	p-value
IGHL anterior band-humeral	2.871	0.035	2.303	0.042	1.049	0.311
IGHL anterior band-glenoid	1.229	0.265	2.958	0.013	1.804	0.091
Axillary recess-humeral	0.634	0.55	1.21	0.252	0.702	0.493
Axillary recess-glenoid	1.12	0.306	1.891	0.085	0.624	0.542
Coracohumeral ligament	-0.56	0.598	-0.285	0.781	-0.023	0.982
Anterior capsule thickness	-0.377	0.719	1.42	0.183	2.905	0.011

Comparison of MRI findings between baseline and the 12-week follow-up demonstrated significant reductions in capsular thickness. Statistically significant improvement was observed in the thickness of the anterior band of the IGHL at both humeral and glenoid attachments, axillary recess measurements, coracohumeral ligament thickness, and anterior capsule thickness, indicating radiological improvement following intra-articular steroid injection (Table 17).

**Table 17:** Overall MRI changes from baseline to 12 weeks. Statistical analysis: Paired t-test. IGHL: inferior glenohumeral ligament.

Parameter	Baseline	12 weeks	t-value	p-value
IGHL anterior band-humeral	7.24 ± 1.34	6.20 ± 0.79	3.22	0.005
IGHL anterior band-glenoid	7.01 ± 1.33	5.84 ± 0.73	3.56	0.002
Axillary recess-humeral	5.04 ± 1.11	4.52 ± 0.90	3.33	0.004
Axillary recess-glenoid	4.78 ± 1.19	4.33 ± 0.86	2.47	0.024
Coracohumeral ligament	2.15 ± 0.60	2.07 ± 0.59	2.22	0.04
Anterior capsule thickness	4.76 ± 0.59	4.34 ± 0.49	3.07	0.007

Shoulder range of motion improved significantly from baseline to the 12-week follow-up in all assessed movements. Significant improvements were observed in internal rotation, external rotation, abduction, adduction, flexion, and extension following treatment (Table 18).

**Table 18:** Overall range of motion changes from baseline to 12 weeks. Statistical analysis: Paired t-test.

Movement	Baseline	12 weeks	t-value	p-value
Internal rotation	38.16 ± 8.85	60.26 ± 10.99	−7.32	<0.001
External rotation	24.21 ± 11.09	56.84 ± 17.66	−8.24	<0.001
Abduction	36.32 ± 12.57	71.84 ± 20.22	−7.51	<0.001
Adduction	14.21 ± 4.17	24.74 ± 6.34	−7.98	<0.001
Flexion	52.37 ± 20.84	97.63 ± 22.81	−6.90	<0.001
Extension	16.84 ± 5.82	32.37 ± 8.23	−7.69	<0.001

Among patients who were in clinical Stage 3 at enrollment, follow-up MRI demonstrated significant reductions in the thickness of the IGHL anterior band at both humeral and glenoid attachments and in anterior capsule thickness. Changes in axillary recess measurements and coracohumeral ligament thickness did not reach statistical significance (Table 19).

**Table 19:** MRI changes in Stage 3 patients. Statistical analysis: Paired t-test. IGHL: inferior glenohumeral ligament.

Stage 3 at enrollment (n=6)	Baseline	12 weeks	t-value	p-value
IGHL anterior band-humeral	6.70 ± 0.89	6.17 ± 0.60	3.02	0.029
IGHL anterior band-glenoid	6.60 ± 0.99	5.85 ± 0.51	2.88	0.035
Axillary recess-humeral	4.10 ± 0.50	3.85 ± 0.36	2.03	0.098
Axillary recess-glenoid	3.80 ± 0.51	3.62 ± 0.37	2.02	0.1
Coracohumeral ligament	2.55 ± 0.70	2.50 ± 0.68	2.24	0.076
Anterior capsule thickness	4.37 ± 0.33	4.20 ± 0.30	5	0.004

Patients presenting with Stage 2 adhesive capsulitis showed significant reductions in MRI measurements following treatment. Significant improvement was observed in the thickness of the IGHL anterior band, axillary recess, and anterior capsule, whereas changes in coracohumeral ligament thickness were not statistically significant (Table 20).

**Table 20:** MRI changes in Stage 2 patients. Statistical analysis: Paired t-test. IGHL: inferior glenohumeral ligament.

Stage 2 at enrollment (n=11)	Baseline	12 weeks	t-value	p-value
IGHL anterior band-humeral	8.03 ± 0.68	6.21 ± 0.86	6.25	<0.001
IGHL anterior band-glenoid	7.72 ± 0.79	5.84 ± 0.86	5.41	<0.001
Axillary recess-humeral	5.72 ± 0.90	4.92 ± 0.94	3.56	0.005
Axillary recess-glenoid	5.50 ± 1.00	4.69 ± 0.90	3.29	0.008
Coracohumeral ligament	1.99 ± 0.50	1.89 ± 0.47	2.06	0.067
Anterior capsule thickness	5.16 ± 0.30	4.48 ± 0.57	3.44	0.006

Among patients who were in Stage 1 at enrollment (n=2), no statistically significant changes were observed in any of the MRI parameters between baseline and the 12-week follow-up. Although numerical increases were noted in the thickness of the anterior band of the inferior glenohumeral ligament (IGHL) at both the humeral and glenoid attachments, axillary recess measurements, and anterior capsule thickness, none of these changes reached statistical significance (all p > 0.05). Similarly, coracohumeral ligament thickness remained unchanged throughout the follow-up period (Table 21).

**Table 21:** MRI changes in Stage 1 patients. Statistical analysis: Paired t-test. IGHL: inferior glenohumeral ligament.

Stage 1 at enrollment (n=2)	Baseline	12 weeks	t-value	p-value
IGHL anterior band-humeral	4.50 ± 0.14	6.20 ± 1.41	−1.55	0.366
IGHL anterior band-glenoid	4.35 ± 0.21	5.80 ± 0.99	−1.71	0.338
Axillary recess-humeral	4.10 ± 0.71	4.30 ± 0.71	0	1
Axillary recess-glenoid	3.75 ± 0.64	4.45 ± 0.35	−3.50	0.177
Coracohumeral ligament	1.80 ± 0.00	1.80 ± 0.28	0	1
Anterior capsule thickness	3.75 ± 0.07	4.00 ± 0.14	−1.67	0.344

## Discussion

The present prospective single-arm interventional study evaluated the association between clinical staging and magnetic resonance imaging (MRI) findings in adhesive capsulitis and assessed the effect of intra-articular corticosteroid injection on clinical, functional, and radiological outcomes over a 12-week follow-up period. The study demonstrated a significant association between MRI parameters and clinical stage, with progressive improvement in pain, shoulder function, range of motion, and capsular thickness following treatment. Patients presenting in Stage II demonstrated greater improvements in MRI parameters, shoulder range of motion, pain (VAS), and DASH scores than those presenting in Stage 3, suggesting a more favorable response to treatment in the earlier stages of adhesive capsulitis. Whereas improvement in Stage 3 was comparatively limited and Stage I patients demonstrated variable MRI changes, reflecting the dynamic inflammatory phase of the disease.

Adhesive capsulitis is characterized by progressive inflammation followed by capsular fibrosis, leading to pain and restriction of shoulder motion. Early diagnosis and timely intervention are essential to prevent progression to irreversible capsular contracture and prolonged disability, as emphasized by Vita et al. [1] and Neviaser and Hannafin [7]. Similar to previous reports, the majority of patients in the present study presented during the freezing and frozen stages, reflecting the stage at which patients commonly seek medical attention [3,4,9].

MRI plays an increasingly important role in evaluating adhesive capsulitis because it allows objective visualization of capsular inflammation and fibrosis. In the present study, progressive thickening of the inferior glenohumeral ligament (IGHL), axillary recess, and anterior capsule was significantly associated with advancing clinical stage. These findings are consistent with those reported by Sofka et al. [2], Chellathurai et al. [15], and Park et al. [14], who demonstrated that MRI findings closely parallel clinical staging and disease severity. Likewise, Zappia et al. [8] highlighted the usefulness of MRI in identifying characteristic capsular abnormalities and differentiating adhesive capsulitis from other causes of shoulder pain.

The anterior band of the IGHL and anterior capsule thickness demonstrated significant stage-wise differences and significant improvement following treatment, indicating their potential utility in the assessment of adhesive capsulitis. These structures demonstrated progressive thickening with advancing disease and significant reduction after corticosteroid injection. Similar observations were reported by Ahn et al. [10], who found that capsular thickening correlated with clinical impairment, and by Choi and Kim [16], who demonstrated significant associations between MRI abnormalities and shoulder stiffness in early adhesive capsulitis. More recently, Beraldo et al. [11] also reported a significant association between MRI findings and clinical assessment, supporting the role of MRI as an objective marker of disease severity.

Pain reduction and improvement in upper-limb function were significant throughout the follow-up period. Progressive decreases in Visual Analogue Scale (VAS) and DASH scores were accompanied by substantial improvements in shoulder range of motion following intra-articular corticosteroid injection. Similar clinical improvement has been reported following corticosteroid therapy and structured rehabilitation programs [7,12,17,21]. Pain relief following intra-articular corticosteroid injection may have facilitated better adherence to the prescribed home-based exercise program, which could have contributed to the observed improvements in shoulder function and range of motion.

Serial MRI demonstrated significant reductions in the thickness of the IGHL, axillary recess capsule, and anterior capsule over the 12-week follow-up period, paralleling improvements in pain and shoulder mobility. These findings support the hypothesis that MRI changes reflect true structural recovery rather than symptomatic improvement alone. Similar reductions in capsular abnormalities following treatment have been described by Park et al. [14], Chellathurai et al. [15], and Choi and Kim [16]. In contrast, coracohumeral ligament thickness showed minimal interval change, consistent with previous observations suggesting that this parameter is more useful for diagnosis than for monitoring disease progression or treatment response [20].

An important finding of the present study was that patients treated during Stage 2 demonstrated the greatest radiological and clinical improvement. Stage 3 patients also improved but exhibited more persistent capsular thickening, whereas Stage 1 patients showed variable MRI changes, likely owing to the evolving inflammatory process and the limited sample size. These observations suggest that Stage 2 represents the optimal therapeutic window for intra-articular corticosteroid injection, a finding that is supported by previous clinical and imaging studies [1,14,15,17,21].

The present study has important clinical implications. MRI not only assists in confirming the diagnosis of adhesive capsulitis but also provides an objective assessment of disease severity and response to treatment. Measurement of the anterior band of the IGHL and anterior capsule thickness appears particularly valuable for disease staging and follow-up. Combining MRI findings with clinical assessment may facilitate individualized treatment planning, improve patient counseling, and identify patients who may benefit from additional interventions such as hydrodilatation or arthroscopic capsular release [2,8,10,11,14].

The strengths of this study include its prospective design, standardized MRI protocol, serial clinical and radiological assessment, and simultaneous evaluation of pain, disability, shoulder motion, and MRI parameters. The association of MRI findings with clinical staging before and after intervention provides a comprehensive assessment of disease progression and treatment response.

Limitations

The study was conducted at a single tertiary-care center. The sample size was estimated using a prevalence-based approach rather than a formal power analysis for the primary outcome, which limited generalizability. Follow-up was limited to 12-14 weeks, preventing assessment of long-term structural recovery. The small number of Stage 1 patients limited the statistical power of subgroup analysis, and the inter-observer reliability of MRI measurements was not evaluated. Larger multicenter studies with longer follow-up are required to validate these findings and further establish MRI-based staging and prognostic assessment in adhesive capsulitis.

Overall, the present study demonstrates a strong clinico-radiological association in adhesive capsulitis and confirms that MRI is a valuable adjunct to clinical evaluation for disease staging and monitoring treatment response. Early intra-articular corticosteroid injection, particularly during Stage 2 disease, appears to provide the greatest structural and functional benefit, supporting the role of timely diagnosis and stage-specific management.

## Conclusions

This prospective single-arm interventional study demonstrated significant stage-wise differences in MRI findings and significant clinical, functional, and MRI improvements during follow-up after intra-articular corticosteroid injection in patients with adhesive capsulitis. The findings suggest that MRI may serve as a useful adjunct to clinical assessment by providing objective information on stage-related structural changes and monitoring changes observed during follow-up. However, owing to the absence of a control group, the observed improvements cannot be attributed solely to the intervention and may also reflect the natural course of the disease. Furthermore, the relatively small sample size and single-center design limit the generalizability of the findings. Therefore, the results should be interpreted with caution, and larger multicenter randomized controlled studies with adequate statistical power are required to further establish the role of MRI in the staging and longitudinal assessment of adhesive capsulitis.
